# Parental involvement in decision‐making about their child’s health care at the hospital

**DOI:** 10.1002/nop2.180

**Published:** 2018-07-30

**Authors:** Antje Aarthun, Knut A. Øymar, Kristin Akerjordet

**Affiliations:** ^1^ Department of Paediatrics Stavanger University Hospital Stavanger Norway; ^2^ Faculty of Health Sciences University of Stavanger Stavanger Norway; ^3^ University of Bergen Bergen Norway; ^4^ School of Psychology, Faculty of Social Sciences University of Wollongong NSW Australia

**Keywords:** coping, decision‐making, health promotion, paediatric, parent involvement, parent role

## Abstract

**Aim:**

To explore parents' experiences on parental involvement in decision‐making about their child's health care at the hospital and to identify how health professionals can improve parental involvement.

**Design:**

An explorative descriptive qualitative study within a constructivist research paradigm.

**Methods:**

Individual semistructured interviews were conducted with a purposive sample of 12 parents. Qualitative content analysis was performed.

**Results:**

This study gives unique insight into how parental involvement in children's healthcare decisions influence parents' ability to cope with the parental role at the hospital. The results showed that parents' competence and perceived influence and control over their child's health care appeared to affect how they mastered their role of involvement in decision‐making. Individually tailored and respectful facilitation of parental involvement in these decisions by health professionals seemed to improve parents' influence, control and ability to cope with the parental role. Nurses should thus strengthen parents' sense of coherence enhancing the quality of health care.

## 1 INTRODUCTION

In many Western countries parents have a legal right to participate in decision‐making (DM) about their child's health care to ensure that health care is provided in accordance with the children's and the families' needs and preferences (Entwistle & Watt, 2006; Thompson, 2007). From a health promotion perspective, this provides parents the opportunity to improve their personal control over their child's health care and their own life circumstances (Eriksson & Lindström, 2008). This is in line with the World Health Organization's (WHO) health promotion strategy, which recommends supportive environments and implementation of salutogenesis in societies (Eriksson & Lindström, 2008; WHO, 2009). The theory of salutogenesis is about peoples' dispositions and resilience to face life and its challenges (Antonovsky, 2012). Salutogenesis focuses on factors that promote health and the ability to cope by facilitating people's sense of coherence; enhancing their perception of life as meaningful, comprehensible and manageable. According to this strategy health professionals (HPs) can strengthen parents' sense of coherence when involving parents in children's healthcare decisions by that is, clarifying their legal rights, treatment options and daily caring routines.

In Norway as in most Western countries, parents are user representatives of their children until their children can fully represent themselves (Patients' Rights Act, 1999). As the main guiding principle, parents are responsible of giving consent to health and medical examinations and treatments on behalf of their child until they are 16 years old. In addition, parents have a legal right to participate in DM to customize their child's health care. This implies that parents have the opportunity to be involved in and influence the DM concerning individual modifications to their child's care, examinations and treatments. This is in line with family‐centred care approaches, which expect parents to participate in partnership with HPs in the coproduction of children's health care (Smith, Swallow, & Coyne, 2015). Parents have valuable knowledge about their child and are important helpers in implementing their children's health care (Harrison, 2010; Watts et al., 2014). Increased parental involvement in DM about children's health care is expected to increase the individual customization of children's health care and thereby improve the quality of care and safety (Ministry of Health & Care services, 2009).

Although parental involvement in decisions about their child's health care is widely acknowledged, parents do not participate as much as they would like to (Aarthun & Akerjordet, 2014; Foster, Whitehead, & Maybee, 2010). In addition, they seem to be in a particularly vulnerable situation when participating in these DM processes. Moreover, this new conceptualization of parental involvement has led to significant changes in the role of both HPs and parents (Aarthun & Akerjordet, 2014), which may be challenging to implement in clinical settings. There is thus a need to explore current practice on parental involvement in DM to gain increased knowledge about parents' role as user representatives of their children.

### 1.1 Background

Patient involvement in health services‐related DM is a complex concept and includes several approaches (Entwistle & Watt, 2006; Thompson, 2007). One main approach focuses on the patient‐professional interaction and patients' degree of involvement and influence during the DM process (Wirtz, Cribb, & Barber, 2006). The shared DM model is a part of this approach were the parents and the HPs are expected to share information and reach consensus (Kon, 2010). This model is relevant when parents participate in DM concerning the customizing and preparation of their child's health care. However, the parents' influence is restricted by HPs' responsibility of giving a health care that is justifiable and within the hospital's framework (Patients' Rights Act, 1999). Another DM approach focus on parents' cognitive and emotional information processing, where psychosocial factors and health literacy are important aspects (Edwards, Davies, & Edwards, 2009; Entwistle & Watt, 2006). Health literacy refers to the essential cognitive and social skills parents need when acquiring knowledge and using information to make decisions about their child's health and health care (Nutbeam, 2009).

Previous studies report that parents want to be involved in decisions about their child's health care to varying forms and degrees of involvement and this desire may change over time (Aarthun & Akerjordet, 2014). Their preference of involvement seems to depend on factors such as parents' demographic characteristics (e.g., age, level of education, income and marital status), emotional condition and competence (Aarthun & Akerjordet, 2014; Jackson, Cheater, & Reid, 2008; Lipstein, Brinkman, & Britto, 2012). Other influencing factors are type of illness, whether the illness is acute or chronic, the seriousness of the condition and parents' prior experiences with health service (Lipstein et al., 2012). Health‐related decisions have, however, become more complex because of enhanced multidisciplinary practice and increased advanced treatment methods (Lipstein et al., 2012; Ofstad, Frich, Schei, Frankel, & Gulbrandsen, 2014). Many parents have limited understanding of illness, treatment and how health services function (Corlett & Twycross, 2006). Moreover, several parents experience emotional distress because of their child's health condition, which may hinder their involvement (Jackson et al., 2008; Tallon, Kendall, & Snider, 2015). Accordingly, parents seem to be in a particularly vulnerable situation in their role as user representatives of their children. Mainly, having a need for support from professionals when being involved in their child's healthcare decisions (Aarthun & Akerjordet, 2014). However, it varies whether and how HPs involve parents in these decisions (Aarthun & Akerjordet, 2014).

There is scarce knowledge about parents' role and needs in terms of their involvement in DM about preparing children's health care in hospitals (Aarthun & Akerjordet, 2014; Lipstein et al., 2012; Shields et al., 2012). In our research, this is considered as an interdependent process, which includes information exchange, discussions, deliberations and reaching consensus using the shared DM model. An increased understanding of the challenges and needs of parents concerning their involvement in their child's healthcare decisions has the potential to give important knowledge and implications for clinical practice.

### 1.2 Objective

The objectives of this study were to explore parents' experiences on parental involvement in DM about their child's health care at the hospital and to identify how HPs can improve parental involvement.

## 2 THE STUDY

### 2.1 Design

This study used an exploratory descriptive qualitative design within a constructivism research paradigm, an interpretive approach (Lincoln, Lynham, & Guba, 2013). Semistructured interviews were used to generate data about the informants' descriptions of their experiences (Peräkylä & Ruusuvuori, 2013). According to the research paradigm, interviews are considered complex social performances where both the interviewer and the informants are active contributors in coconstructing the informants' account of their experiences (Silverman, 2011).

### 2.2 Method

#### 2.2.1 Selection of informants

A purposive selection procedure was applied to select informants at the Department of Paediatrics of a university hospital in Norway (Silverman, 2013). New informants were included up to saturation (N + 1), meaning that when sufficient data had been obtained and no new variations in knowledge appeared, only one more interview was performed (Daly et al., 2007). This resulted in 12 informants. The inclusion criteria were individuals with parental responsibility for a child who was staying or had stayed in a paediatric ward at the hospital in the last 3 months. The parents also needed to have sufficient fluency in Norwegian to participate in the interview. In In addition, the sample should represent parents of both genders, parents of children ranging in age from newborn to 16 years and parents who had been admitted to different paediatric wards within the hospital. Clinical nurses at the three different paediatric inpatient wards recruited the informants.

#### 2.2.2 Data collection and setting

The interviews were conducted from February to September 2014. The data were collected in one individual semistructured interview per informant that was audio recorded (Ryan, Coughlan, & Cronin, 2009). The interview guide was based on a systematic review (Aarthun & Akerjordet, 2014) and the theory of salutogenesis (Antonovsky, 2012) and user involvement (Entwistle & Watt, 2006; Thompson, 2007). Two of the authors agreed on the included questions. Typical questions to the informants were: “Please tell me about your child's last admission to the hospital” and “How were you involved in DM about preparing your child's healthcare?” Ten informants were interviewed during their child's hospitalization, one was interviewed 4 days later at the hospital and the other 7 days later at the informant's workplace. The interviews lasted between 35 and 90 min. After the interviews, the informants answered a survey with demographic questions that gave information on their background (Table 2). In addition, the interviewer made notes about the interview setting. The recorded interviews were transcribed verbatim, with the exception of identifying details, which were anonymized or removed.

The department of paediatrics offered health care to children from the ages of 0–16 years and has a neonatal ward, an infection ward and a general medical ward. Approximately 3,500 children are hospitalized annually and 13,000 receive outpatient consultations each year. Interprofessional cooperation is emphasized at the department level, meaning that individuals in different health professions, such as registered nurses, physicians, physiotherapists and dietitians, work closely in teams. They collaborate in the DM regarding the children's health care. In addition, individuals in each profession are responsible for involving parents in the aspects of the children's care plan that fall in their subject area.

### 2.3 Analysis

To facilitate the organization of data, the transcripts were entered into the data management system NVivio 10 for manual coding (Bazeley & Jackson, 2013). Two of the authors (AA and KA) performed the analysis according to the qualitative content analysis described by Graneheim, Lindgren, and Lundman (2017), Graneheim and Lundman (2004). Initial coding and the identification of preliminary categories was performed by AA. Further analysis was discussed with KA and the authors reached a consensus on the final composite analysis. First, the transcripts were read several times to give an impression of the parents' experiences of the parental role and involvement in DM about their child's health care in the hospital. Second, relevant transcripts were extracted and divided into meaning units which are sentences that contain a central meaning related to the context (Graneheim & Lundman, 2004). The condensed meaning units were then coded and compared to examine similarities and differences. This manifest content analysis resulted in a set of subcategories and categories. Third, after comparison and interpretation of the manifest categories, one main theme and two subthemes were identified that reflected the latent content of the transcripts; a higher level of data interpretation. Table 1 gives information from the analysis process.

**Table 1:** Examples from the analysis based on Graneheim and Lundman (2004)

Meaning unit	Condensed meaning unit	Category	Subtheme	Main theme
“Sometimes it's difficult to judge a recommendation because you think you are not competent. Then, you think they (health professionals) are so competent and have done it before.”	The parent thought that he sometimes did not have enough competence to be active involved in decision‐making and that the health professionals were so competent.	Parental competence and understanding.	Parental competence and need for information.	A demanding parental role.
“You become involved and informed and you can calm yourself because you understand that they are doing what is best for your child.”	Parental involvement in decision‐making increase parents' sense of security and control of their child's health care.	Parental involvement. Parental influence and control.	Parental involvement and control.	

#### 2.3.1 Methodological considerations

The researchers conducted this qualitative study according to the constructive research paradigm aiming scientific rigour and trustworthiness (Carter & Little, 2007; Graneheim et al., 2017). This was influenced by the researchers preunderstanding and context, culture and time (Altheide & Johnson, 2013). All authors had a professional preunderstanding as experienced clinicians in various fields such as paediatric physiotherapy, critical care nursing and paediatric medical practice in hospital settings.

The interviewer was a paediatric physiotherapist who was familiar with the hospital wards, which increased the understanding of the informants' descriptions of the context (Silverman, 2011). The notes describing the interview settings gave valuable additional information about the informants' role and the context during the interviews. The applied research paradigm assume that the findings are a product of the researchers' interpretations of the informants' accounts of their experiences as situated in time (Allen & Cloyes, 2013; Choen & Crabtree, 2008), accordingly the informants were not asked to confirm the findings.

### 2.4 Ethics

The study adhered to the general ethical principles outlined in the Declaration of Helsinki (World Medical Association, 2013). All informants received both oral and written information about the study. They were also informed about the voluntary nature of participation and the ability to withdraw from the study at any time and were guaranteed confidentiality. The informants gave written informed consent to participate in the study.

## 3 RESULTS

### 3.1 The sample

The demographics of the informants are presented in Table 2. The sample consisted of four parents from each of the three paediatric wards. One parent was nonnative Norwegian and one was married to a nonnative. The informants' children were aged from being newborn to 11 years old, with various healthcare needs.

**Table 2:** The demographics of the informants

Nr.	Gender	Age	Diagnosis of child	Number of children	Number of child's hospitalization	Education level
1	Female	36	Cancer	3	>5	Upper secondary education
2	Female	39	Premature	2	1	College/university (1–4 years)
3	Male	35	Lung disease	2	>5	Upper secondary education
4	Male	41	Lung disease	2	2	College/university (5 years or more)
5	Female	47	Evaluation process for diagnosis	3	1	College/university (1–4 years)
6	Female	35	Premature	1	1	Upper secondary education
7	Female	41	Evaluation process for diagnosis	2	3	College/university (5 years or more)
8	Female	40	Heart failure	2	4	College/university (5 years or more)
9	Female	35	Immune deficit	3	>5	Upper secondary education
10	Female	28	Premature	1	1	College/university (1–4 years)
11	Female	32	Evaluation process for diagnosis	1	1	College/university (1–4 years)
12	Female	24	Premature	1	1	College/university (1–4 years)

### 3.2 Identified themes

The main theme, “a demanding parental role”, was identified with two subthemes: “parental competence and need for information” and “parental involvement and control”. The parents were highly concerned about their child's health care and perceived their parental role as user representatives of their child in the hospital to be demanding. This was particularly when the parents felt lack of continuing of health care, which led to increased stress, concern and insecurity. Mastery of involvement in decisions about their child's health care, seemed to depend on parental competence and how parents perceived their influence and control in DM. HPs' facilitation of parental involvement in DM and provision of sufficient and consistent information appeared to be of great importance. This indicate that the HP's role was essential in facilitating parents' ability to cope with the parental role during children's hospitalization by promoting parents' ability to perceive their role as meaningful, comprehensible and manageable. The subthemes are presented below and describe the parents' challenges, needs and preferences in mastering the parental role of involvement in DM about their child's health care at the hospital.

#### 3.2.1 Parental competence and need for information

Lack of parental competence and insufficient information from the HPs appeared to make the parental role of involvement in DM demanding. This was related to the parents' knowledge and understanding of their child's health condition, needs and health care, which depended on their previous experiences, perceived stress and respect for the HPs' competence. In addition, some parents' lack of knowledge about the Norwegian health services and culture seemed to negatively influence their involvement in their child's healthcare decisions.

Generally, parents stated that they had little healthcare knowledge even if the HPs had provided a substantial amount of information. This made it difficult for the parents to participate in decisions about their child's health care, particularly in decisions about the individual preparing of medical examinations and treatments. One mother said the following:

We think that you ought to have so much information, but at the same time, you know so little. Thus, as parents, we have to trust that the HPs know what they do and believe that they do what's best for the child. (8)

Several parents experienced a lack of knowledge about their child's health condition, disease and needs, which affected their ability to participate in influencing their child's health care. They needed to receive much more information from HPs before they could actively participate in DM. Thus, lack of knowledge influenced their comprehensibility and manageability. In the initial stages of their hospital stay, parents therefore preferred for the HPs to give clear recommendations about their child's health care. However, when the parents acquired more knowledge of their child's special needs and increased their own experiences in assisting with different healthcare settings, they became more capable of participating in determining their child's health care. They then took a greater role in discussions about their child's health care. The parents also perceived receiving different options about their child's health care more positively because they were better able to judge the various possibilities. One mother expressed the following:

It's nice to hear different perspectives, but it can also be very confusing. It can be a bit frustrating and stressful when a HP says, e.g., using breastplates doesn't influence the child's suckling, while others say you will affect ordinary breastfeeding because it presents another technique. In the beginning, you get frustrated, but as time goes by, you have to decide yourself …. (10)

Parents with long‐term ill children who had acquired a substantial amount of experience and knowledge about their child's condition, needs and health care expressed this notion in particular. These parents were more actively involved in DM about preparing their child's health care. Other parents emphasized the fact that despite the availability of good information, they did not achieve sufficient understanding of their child's condition to participate in DM due to a high degree of distress. In particular, this was difficult for parents with critically ill children. One mother said the following:

You get a depressing message and it worsens over a period of time when you feel broken. You're not capable of participating in DM. (1)

Some parents received incomplete, incomprehensible or inconsistent information about their child's health condition, needs and health care from the HPs, especially when parents felt lack of continuity and coordinated health care. Thus, they became confused, frustrated and insecure, not knowing which of the professionals they should listen to. This made it difficult to achieve sufficient insight and comprehensibility of their child's condition and needs and thus too demanding to take an active role in determining their child's healthcare plans. An example which illustrates this was one mother who expressed:

When you have a new (nurse) in the morning, a new one in the afternoon and a new in the evening, so there are three persons during 24 hours and when there are three new nurses the next day and three after that… you get confused about who is who and who has said what and who you should listen to because the nine persons are very different and have their own opinions about different things. (12)

Moreover, some parents seemed to have a limited knowledge and understanding of the Norwegian healthcare services, for example, some parents experienced that they did not behave according to HPs' expectations when implementing their child's care. Cultural differences and lack of information from the HPs seemed to lead to misunderstandings in the communication with HPs reducing parents' comprehension of their child's healthcare services. This negatively affecting the parents' involvement in DMs and thereby their manageability of the parental role.

#### 3.2.2 Parental involvement and control

There was considerable variation in how and the extent to which the parents perceived they were involved in decisions about their child's health care. Several parents perceived a lack of influence and control in their child's health care, making the parental role as user representative demanding. Furthermore, HPs' facilitation of parental involvement in DM seemed to influence how the parents perceived their level of control, influence in decisions and empowerment. This indicates that HPs' facilitation of parental involvement influenced parents' manageability of the parental role. One mother described her opportunity to be involved in DM about her child's care as the following:

How much parents are involved in DM about their participation in care is often dependent on the nurse. Some ask you what you want to do today to care for the child. Do you want to do this or this? Have you thought about this? Do you want to try this? Maybe we ought to do this some days? However, some nurses don't involve you and just administer the care of the child. (12)

Some parents experienced lack of being involved in decisions or a sense of not being listened to by HPs, which led to powerlessness, insecurity and little self‐confidence when they sought health care for their child. One mother expressed it as such:

One of the worst things you can experience as a mother is having to explain the same things several times and not being listened to. You sit there and feel so powerless. (7)

To enhance parental influence and control on their child's health care, it was thus of utmost importance that HPs promoted parents' involvement in DM about their children's health care. This required HPs to provide parents with improved opportunities to gain an understanding of their child's health condition, needs and health care through sufficient, consistent and individual tailored information. As a result, parents became convinced that their child was receiving the right form of health care which improved their sense of security and control of the situation. In addition, they became more active involved in the DM process. One father stated the following:

Being involved obviously makes us feel certain about what's happening. We can understand it better when we participate and discuss the progress. Is it becoming worse? Is it getting better? Should we do things differently? (3)

Furthermore, parents who received support about the importance of their knowledge and opinions were of significance felt that they influenced their child's health care. This positive experience facilitated an active seeking of information and parental involvement in DM, enhancing their empowerment. One father expressed this as follows:

If you receive support about the importance of your point of view, it can be an incentive that helps you become more active and further investigate the situation. When HPs involve you in preparing your child's health care and give you information, they are providing an opportunity to participate more actively. Parents then feel more empowered. (4)

This indicates that HPs' facilitating of parental involvement in DM's promoted parents' manageability and comprehensibility of their child's health care. The opportunity to be involved in preparing their child's health care was especially important to parents of long‐term seriously ill children. Although it was both demanding and informative, the parents needed support from the HPs on their opinions about their child's health care and their performance of the parental role. This helped the parents take responsibility and manage severe stress over time.

However, several parents who were involved in preparing their child's health care struggled to ask for help to address their own needs, wishes and preferences. In these circumstances, it was easier for the parents to express their own needs and opinions when they had regular conversations with the HPs, particularly when the professionals showed genuine concern for the parents' situation. This indicated that the parents preferred being involved in preparing their child's health care through regular conversations with well‐known HPs. One mother expressed this as follows:

Take us out of the ward to discuss what we think about our child's health care, what has happened, what we're wondering and ask us if there is something we need or would prefer. Just as an evaluation of the hospital stay. Then, they'll get to know what we're dissatisfied with or very pleased about and then they can carry that information on to the other HPs. (10)

## 4 DISCUSSION

The findings indicate that parents were highly concerned about their child's health care and were in a very challenging and vulnerable situation during involvement in decisions about their child's health care. Parents' ability to cope in these DM seemed to depend on their competence and how they perceived their influence and control in DM. However, HPs' facilitation of parents' active involvement in these decisions and provision of sufficient and consistent information seemed to empower the parents and increase their active involvement in DM. Accordingly, the parents' ability to cope with the parental role in the hospital appeared to be strengthened by promoting their perception of life as meaningful, comprehensible and manageable; their sense of coherence, when involving parents in children's healthcare decisions.

The findings extend previous research on parental involvement in DM concerning children's health care from a health promotion perspective. The findings, that is, a demanding parental role, the significance of parental competence and understanding and the importance of receiving consistent and sufficient information from HPs, confirm previous research (Aarthun & Akerjordet, 2014; Corlett & Twycross, 2006; Foster et al., 2010). However, this study contributes new insight into parents' role as user representative of their children which seems to be an important aspect of parents' ability to cope with the parental role in the hospital (Antonovsky, 2012). The findings also highlight HPs' essential role in both facilitating parents' active involvement in children's healthcare decisions and in improving parents' ability to cope with their parental role during hospitalization. In this regard, HPs are important contributors to the provision of health promotion, which should be more emphasized in this context.

In line with previous research, our study shows that parents need a substantial amount of information about their child's health condition, disease and the healthcare system to be able to participate in decisions related to their child's health care (Jackson et al., 2008; Power & Franck, 2008; Uhl, Fisher, Docherty, & Brandon, 2013). Parents with more experience from their child's hospitalization had a good understanding of their child's condition and the healthcare system and were more actively involved in preparing their child's health care (Lipstein et al., 2012). Nonetheless, it was still difficult for them to participate in decisions about the medical component of health care due to a lack of knowledge (Power & Franck, 2008; Uhl et al., 2013). Furthermore, parents with a limited knowledge of the Norwegian health services and culture appeared to have more difficulty communicating and cooperating with the HPs. These factors are reported in the literature on patient's health literacy, which also seems to be an important factor affecting parental knowledge and understanding of their child's condition and health care (Nutbeam, 2009; Sorensen et al., 2015). HPs should therefore become more aware of parents' health literacy and need of individual facilitation when involving parents in their child's healthcare decisions.

Nevertheless, our results indicate that parents are dependent on *if*,* how* and *when* HPs involve them in DM about their child's health care, as supported by the literature (Aarthun & Akerjordet, 2014). This reflects the asymmetry in authority and power between HPs and parents. Moreover, how HPs' involve parents in DM is dependent on many factors such as lack of resources, time constraints and organizational shortcomings as well as HP's attitudes and routinized thinking towards the parental role and their professional role at the hospital (Aarthun & Akerjordet, 2014). There was a considerable variation in the extent to which parents were involved and able to influence their child's health care. Some parents were not involved or listened to and thus felt powerless and uncertain about their child's health care. This seemed to heighten these parents' stress in an already demanding situation and can limit their coping with the parental role in the hospital (Edwards et al., 2009; Tallon et al., 2015). On the other hand, our findings support the notion that HPs' active involvement of parents in their child's healthcare decisions increases parents' competence and engagement in preparing their child's health care (Aarthun & Akerjordet, 2014; Uhl et al., 2013). These findings imply that active involvement and support from HPs enhance parents' influence and control over their child's health care. In addition, the findings indicate that inter‐ and intraprofessional coordination of children's health care is of significance to achieve consistent information to parents. Thus, HPs should become more conscious about how they convey information and involve parents in children's healthcare decisions as a healthcare team. Several parents reported that they preferred to be involved in decisions about their child's health care through regularly appointed conversations with known HPs (Coyne & Cowley, 2007; Roets, Rowe‐Rowe, & Nel, 2012). This gives parents an opportunity to give feedback about their child's health care and their hospital stay. Furthermore, parents who were extremely distressed because of their child's health condition seemed to have unique needs, such as individually tailored facilitation of their involvement in DM concerning their child's health care (Edwards et al., 2009; Power, Swartzman, & Robinson, 2011). This requires HPs to have a high degree of empathy to actively listen to the parents' thoughts, opinions and preferences to improve their involvement and ability to cope with their parental role (Eriksson & Lindström, 2008; Halpern, 2014).

### 4.1 Limitations and further research

The study's inclusion criteria were met. The sample, however, consisted of few males and no single parents, which is a potential limitation. Nevertheless, quantitative studies are required to confirm the results (Polit & Beck, 2010). Qualitative research is needed to improve the understanding of HPs' role in facilitating parental involvement in DM. Further, more research is required to explore how children are integrated in healthcare DM (e.g., their thoughts, wishes and opinions) and how this influence parental involvement during hospitalization. Finally, further knowledge is needed on the parental involvement in DM amongst migrant parents with language and cultural barriers.

## 5 CONCLUSION

This study gives unique insight into parents' perspectives on their parental role as user representative of their children at the hospital primarily from a health promotion perspective. In particular, it expands on the literature on how parental involvement in children's healthcare decisions influence parents' ability to cope with the parental role at the hospital.

Nurses and other HPs should thus safeguard individualized and respectful facilitation of parental involvement in preparing children's health care to strengthen parent's sense of coherence. In addition, to ensure the quality and provision of family‐centred care during children's hospitalization.

## CONFLICT OF INTEREST

The authors declare no potential conflicts of interest with respect to the research, authorship and publication of this article.

## AUTHOR CONTRIBUTIONS

All authors have agreed on the final version and meet at least one of the following criteria [recommended by the ICMJE (https://www.icmje.org/recommendations/)]:

- substantial contributions to conception and design, acquisition of data or analysis and interpretation of data;
- drafting the article or revising it critically for important intellectual content.
